# Computer-aided detection of equivocal spinal tuberculosis on X-ray using a YOLOv11-based deep learning model

**DOI:** 10.3389/fpubh.2026.1780946

**Published:** 2026-06-10

**Authors:** Yan Yuan, Juan Ma, Haiting Ma, Mei Zhang, Xinyue Qiu, Lingyan Shen, Zhenwei Ren, Jiangbin Wang, Adina Abulizi, Wei Hu, Mayidili Nijiati

**Affiliations:** 1 Department of Radiology, Medical Imaging Center, Xinjiang Medical University Affiliated Fourth Hospital, Urumqi, China; 2 Department of Deepwise AI Lab, Hangzhou Deepwise & League of PHD Technology Co., Ltd., Hangzhou, China; 3 Department of Spine Surgery, Xinjiang Medical University Affiliated Fourth Hospital, Urumqi, China; 4 Xinjiang Key Laboratory of Artificial Intelligence Assisted Imaging Diagnosis, Kashi, China; 5 Department of Radiology, The First Affiliated Hospital of Xinjiang Medical University, Urumqi, China

**Keywords:** deep learning, object detection, Pott's disease, referral support, spinal tuberculosis, X-ray imaging

## Abstract

**Background:**

Spinal tuberculosis (STB), also known as Pott's disease or tuberculous spondylitis, remains a substantial clinical burden in some remote and resource-limited regions, where X-ray imaging is often the primary modality available for initial assessment. However, early- and mid-stage STB frequently presents with subtle, equivocal, or atypical structural changes on X-ray images, which may lead to delayed recognition and referral in primary-care settings. Investigating suspicious-region localization in cases with clinical suspicion of STB but equivocal X-ray findings may provide supportive information for further imaging evaluation and referral decisions in primary-care settings.

**Methods:**

This retrospective study included 307 patients from three tertiary hospitals, all of whom had equivocal X-ray findings and CT-confirmed STB. CT-referenced suspicious-region annotations were established on X-ray images according to the CT-confirmed involved spinal levels and their anatomical correspondence on radiographs. A single-class suspicious-region localization model was developed based on the YOLOv11 object detection framework, and the dataset was split at the patient level into training and test sets. Model performance was evaluated using mAP@0.5, mAP@0.5:0.95, precision, recall, F1-confidence curves, precision-recall curves, and confidence-related curves. Patient-level detection rate and missed-case rate were further introduced to evaluate case-level alerting performance. To assess the spatial correspondence between CT-referenced annotations and suspicious regions visible on X-ray images, an agreement analysis between X-ray-only blinded annotations and CT-referenced annotations was additionally performed.

**Results:**

On the test set, the YOLOv11 model achieved an mAP@0.5 of 0.7664, an mAP@0.5:0.95 of 0.4520, a precision of 0.8358, and a recall of 0.6215. Threshold-related curves showed a trade-off between precision and recall across different confidence thresholds. At the provisional working threshold of 0.25, the patient-level detection rate was 0.8073, with a missed-case rate of 0.1927, indicating that the model could provide at least one suspicious-region prompt in most CT-confirmed positive test cases. The agreement analysis showed that all 40 randomly selected test cases could be annotated with at least one suspicious region based on X-ray images alone. The mean maximum IoU between X-ray-only blinded annotations and CT-referenced annotations was 0.7542; 34/40 cases reached a maxIoU ≥0.5, and 38/40 cases reached a maxIoU ≥0.3.

**Conclusion:**

This study shows that, using X-ray images alone as input, a deep learning model can provide suspicious-region localization prompts for CT-confirmed STB cases with equivocal X-ray findings. The proposed approach provides a feasible proof-of-concept framework for early risk prompting and referral support for STB in resource-limited settings. Further studies incorporating normal cases and non-tuberculous spinal disease controls, as well as external validation and prospective evaluation, are required to assess its applicability in real-world primary-care settings.

## 1 Introduction

Spinal tuberculosis (STB) is an important form of extrapulmonary tuberculosis and remains a substantial disease burden in developing regions and areas with limited medical resources. Early recognition of STB before obvious vertebral destruction, spinal deformity, or neurological impairment is important for reducing the risk of long-term disability and improving the timing of treatment (1). Computed tomography (CT) and magnetic resonance imaging (MRI) can clearly demonstrate STB-related osseous destruction, endplate erosion, paravertebral soft-tissue involvement, and bone marrow edema, and are important imaging modalities for further characterizing lesion nature and extent. However, in primary-care or resource-limited settings, CT and MRI are often not available at the initial visit, whereas X-ray imaging remains the most commonly used initial imaging examination for patients with spinal symptoms because of its low cost, broad accessibility, and operational simplicity. Therefore, identifying STB-related suspicious clues from routine X-ray images is a key issue in early risk prompting and referral support in primary-care settings.

In the early stage or in cases with mild osseous involvement, STB often lacks typical signs on X-ray images. Such cases may not yet show obvious vertebral body destruction, intervertebral space collapse, kyphotic deformity, or clearly visible paravertebral abscesses; instead, they may present only as low-contrast, poorly demarcated, or mild structural abnormalities. In this study, these findings, defined as suspicious abnormalities on X-ray images that are insufficient to establish a definitive diagnosis of STB based on plain radiographs alone, are uniformly referred to as equivocal X-ray findings. Under such conditions, experienced radiologists may notice suspicious spinal levels, whereas junior physicians or primary-care clinicians may have difficulty making a confident judgment. By contrast, CT can usually depict mild cortical bone destruction, endplate erosion, and vertebral margin irregularity more clearly, while MRI is more sensitive to bone marrow edema and inflammatory reactions. Therefore, a clinical situation may arise in which X-ray findings are equivocal but CT or MRI evidence is relatively definite, constituting an important obstacle to early recognition and timely referral of STB (2, 3).

This problem is particularly pronounced in resource-limited regions. Many patients with early STB lack specific systemic symptoms, and complaints such as back pain may be attributed to degenerative disease, muscular strain, or other common spinal conditions. When primary-care institutions rely mainly on X-ray imaging for initial assessment, STB cases with equivocal X-ray findings may be identified late, thereby delaying subsequent CT/MRI examination, specialist referral, and initiation of anti-tuberculosis treatment. Thus, an auxiliary tool capable of prompting suspicious regions on X-ray images is needed to help clinicians determine whether further advanced imaging or referral is warranted when clinical suspicion of STB exists but plain radiographs are insufficient for diagnosis.

In recent years, deep learning techniques have been widely applied to chest radiograph analysis, bone disease recognition, and medical image object detection tasks (4, 5). In studies related to spinal infection and STB, existing work has more commonly relied on information-rich tomographic modalities such as CT or MRI for lesion classification, differential diagnosis, or prognostic assessment. These studies provide an important foundation for intelligent imaging analysis of STB, but their application usually presupposes the availability of advanced imaging. In contrast, research on suspicious-region prompting for STB using X-ray images alone in primary-care initial-assessment scenarios remains limited. In particular, for CT-confirmed positive cases with equivocal X-ray findings, it remains insufficiently explored how CT information can be used to establish reasonable suspicious-region annotations on X-ray images and how object detection models can be trained to identify these low-contrast and poorly demarcated regions.

Based on the above clinical need and research gap, this study focuses on a narrower intended-use scenario: primary-care physicians have developed clinical suspicion of STB based on symptoms, medical history, or initial imaging findings, but the X-ray findings remain equivocal and insufficient for a definitive diagnosis based on plain radiographs alone. In this scenario, the role of the model is not to replace clinical diagnosis, nor to screen the general population or all patients with back pain, but to provide STB-related suspicious-region prompts on X-ray images to assist clinicians in judging whether further CT/MRI examination or referral is needed. In this study, CT or MRI findings were used only as structural references for case confirmation and suspicious-region annotation on X-ray images, and were not used as model inputs during training or inference. Accordingly, the proposed framework relies only on X-ray images in its intended application.

To this end, we constructed a multicenter retrospective dataset including 307 CT-confirmed STB cases with equivocal X-ray findings, and established CT-referenced suspicious-region annotations on X-ray images according to the CT-confirmed involved spinal levels. Based on this dataset, a single-class suspicious-region localization model was developed using the YOLOv11 object detection framework. The main objectives of this study were: (1) to construct a CT-referenced suspicious-region annotation dataset for STB cases with equivocal X-ray findings; (2) to evaluate the ability of a deep learning model to provide STB-related suspicious-region localization prompts using X-ray images alone as input; and (3) to analyze the potential application value of the model in early risk prompting and referral support from both region-level and patient-level perspectives.

## 2 Materials and methods

### 2.1 Study cohort construction and case selection

This study was designed as a retrospective, multicenter imaging investigation. Patients were recruited from three affiliated tertiary hospitals of Xinjiang Medical University, with the study period spanning from 2018 to 2024. All three hospitals were equipped with standardized digital radiography (DR) systems and routine spinal CT scanners, and their imaging acquisition protocols and technical conditions showed good consistency. All initial candidate cases consisted of patients who were ultimately diagnosed with spinal tuberculosis (spinal tuberculosis, STB) in routine clinical practice.

The dataset construction strategy in this study was determined by the intended use scenario. In primary-care or resource-limited medical settings, advanced imaging modalities such as CT or MRI are often unavailable at the initial visit, and spinal imaging assessment usually relies first on X-ray examination. Therefore, this study was not intended to perform population-level screening for all patients with back pain or the general population, nor to establish a differential diagnostic model for distinguishing STB from non-tuberculous spinal diseases. Instead, the target scenario of this study was more specific: when primary-care physicians have developed clinical suspicion of STB based on symptoms, medical history, or initial imaging findings, but the X-ray findings remain equivocal and insufficient for a definitive diagnosis based on plain radiographs alone, the model provides early prompts for suspicious regions to support decisions regarding further CT/MRI examination or referral.

Accordingly, only patients with STB confirmed by CT or MRI were included in this study. CT or MRI findings were used as structural references to establish CT-referenced suspicious-region annotations on X-ray images. In addition, CT and MRI information was used only for case confirmation and annotation reference, and was not used as input during model training, validation, or inference. In the intended application scenario, the model analyzes X-ray images alone. Because the current study was designed to address suspicious-region localization and case-level alerting within CT-confirmed STB-positive cases, rather than differential diagnosis in a mixed clinical population, normal controls and non-tuberculous spinal disease controls were not included. This design also means that the present study cannot evaluate model specificity, false-positive burden, or real-world referral yield in mixed clinical populations; these issues require further validation in future studies including normal cases and disease-mimicking conditions.

**Figure 1:**
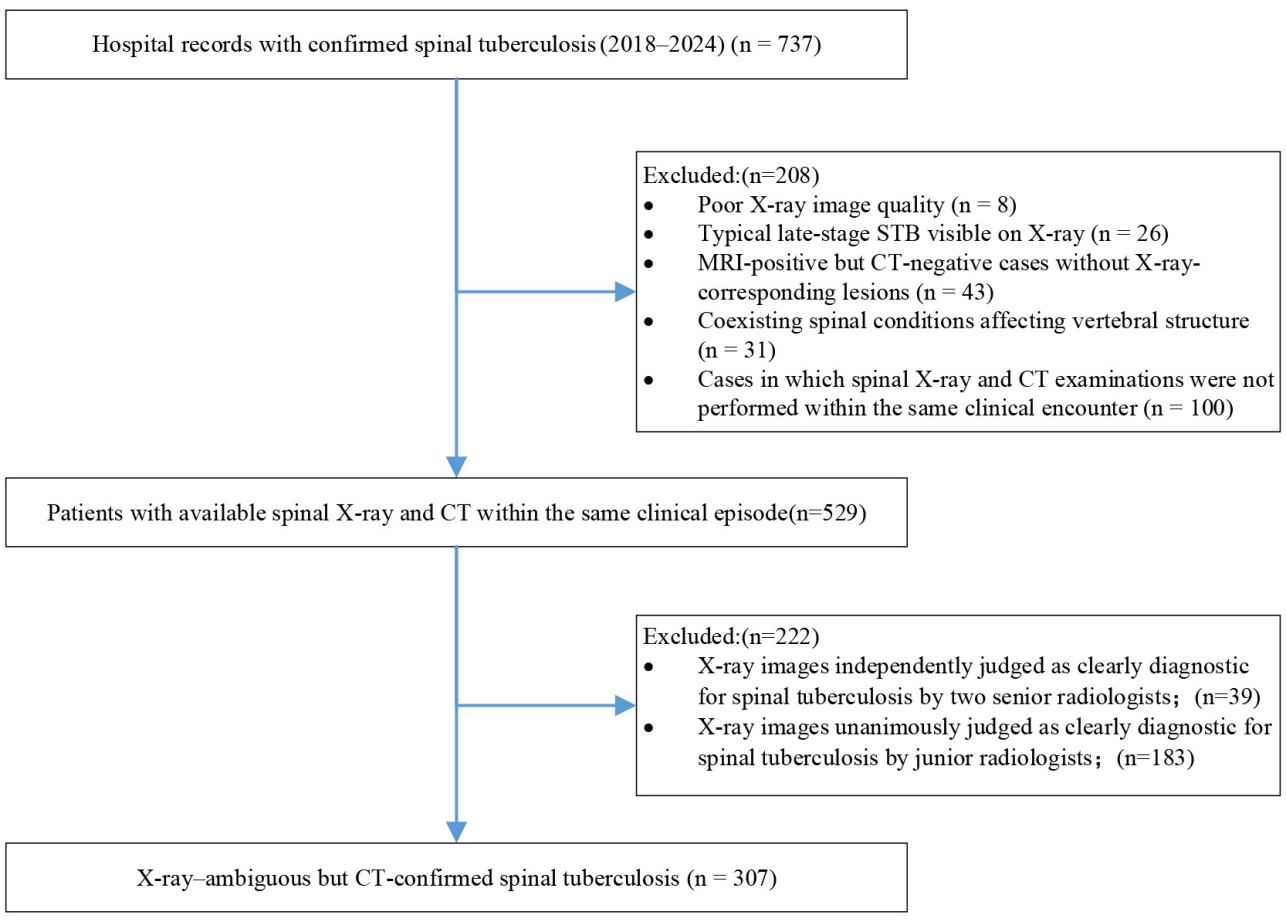
Flowchart of patient inclusion and exclusion.

The diagnosis was established based on a comprehensive assessment integrating clinical manifestations, laboratory findings, CT imaging characteristics, and, when necessary, histopathological evidence. Construction of the study cohort was performed using a stepwise screening strategy, as illustrated in Figure 1.

First, all hospitalized patients diagnosed with STB within the study period were retrieved from the hospital information systems and picture archiving and communication systems, yielding a total of 737 candidate cases. Cases with poor X-ray image quality, including severe underexposure, overexposure, motion artifacts, or incomplete visualization of key vertebral structures, were excluded (*n* = 8). Patients who already exhibited typical late-stage STB manifestations on X-ray images and could be directly diagnosed based on plain radiographs were also excluded (*n* = 26). In addition, cases that were positive only on MRI but negative on CT and for which reliable lesion correspondence could not be established on X-ray images were excluded (*n* = 43). Patients with comorbid conditions that could substantially interfere with vertebral structure assessment, such as pronounced degenerative changes, tumors, or prior traumatic injuries, were removed (*n* = 31). Furthermore, cases in which spinal X-ray and CT examinations were not completed within the same clinical diagnostic cycle were excluded (*n* = 100). After these screening steps, 529 patients remained eligible for subsequent image interpretation and consistency-based selection.

To further focus on cases that posed diagnostic challenges on X-ray imaging, an interpretation-based consistency screening procedure was implemented. In this study, findings defined as suspicious abnormalities on X-ray images that were insufficient to establish a definitive diagnosis of STB based on plain radiographs alone were uniformly referred to as “equivocal X-ray findings." All remaining X-ray images were independently reviewed without reference to CT or MRI findings by four radiologists, including two senior radiologists with more than 10 years of diagnostic experience and two junior radiologists with 2–3 years of experience. Initially, the two junior radiologists independently assessed whether each X-ray image could be definitively diagnosed as STB based solely on plain radiographs. If both junior radiologists considered the image insufficient for a definitive diagnosis under plain radiographic conditions, namely showing equivocal X-ray findings, the case was directly included in the study cohort. For cases with discrepant interpretations between the two junior radiologists, the images were subsequently submitted to the two senior radiologists for independent review. If both senior radiologists judged the X-ray image to be clearly diagnostic, the case was excluded. To reduce the influence of incidental judgment variability, discrepant cases that were not unanimously classified as clearly diagnostic by the senior radiologists were re-evaluated by the two junior radiologists after a 3-week interval. If disagreement persisted upon re-reading, namely if at least one junior radiologist still considered the image insufficient for a definitive diagnosis based on X-ray alone, the case was included in the final study cohort.

Through this consistency-based screening process, a total of 222 cases judged to be clearly diagnostic on X-ray images were excluded, including 183 cases unanimously classified as clearly diagnostic during the initial evaluation by the junior radiologists and 39 cases unanimously classified as clearly diagnostic by the senior radiologists during independent review. All remaining cases with equivocal X-ray findings, suspicious clues, and insufficient diagnostic confidence were retained.

Following the above stepwise screening and multi-level image interpretation procedures, a total of 307 patients meeting the criterion of equivocal X-ray findings but definitive CT positivity were ultimately included, constituting the final analysis cohort of this study. Accordingly, the model was formulated as a single-class object detection task aimed at localizing CT-referenced suspicious regions on X-ray images within CT-confirmed STB cases, rather than performing differential diagnosis between STB and non-tuberculous spinal diseases.

### 2.2 Image acquisition

All patients included in this study were analyzed using standard spinal X-ray images, including anteroposterior or lateral views. Although the specific imaging equipment models differed slightly across institutions, the acquisition parameters remained within routine clinical ranges for spinal imaging. In the routine clinical workflow, patients usually underwent X-ray examination first as the initial imaging assessment; when X-ray images showed suspicious abnormalities or clinical suspicion persisted, CT or MRI examinations were subsequently arranged. According to the clinical practice of the participating institutions, most CT or MRI examinations were performed within several days after X-ray acquisition, or at least within the same clinical diagnostic cycle. Therefore, paired X-ray and CT/MRI images generally reflected similar disease stages and could be used to establish cross-modality anatomical correspondence.

To reduce the influence of disease progression on cross-modality correspondence, this study included only cases in which X-ray and CT examinations were completed within the same clinical diagnostic cycle. CT or MRI findings were used to confirm the involved vertebral levels, the extent of osseous destruction, and adjacent structural changes, and served as spatial references for CT-referenced suspicious-region annotations on X-ray images. It should be emphasized that CT and MRI were used only for case confirmation and annotation reference, and were not involved in model training, validation, inference, or feature extraction. In all training, testing, and intended application stages, the model used X-ray images alone as input.

All three hospitals used digital radiography (DR) systems for spinal imaging. The overall acquisition parameter ranges were as follows: tube voltage of 70–90 kVp, tube current of 20–40 mAs, focus-to-film distance (FFD) of 100–120 cm, and image pixel resolutions of approximately 2, 000 × 2, 000 to 3, 000 × 3, 000. The imaging field of view covered the involved vertebral segments, including thoracic, lumbar, or thoracolumbar regions. All X-ray images were acquired using routine diagnostic exposure parameters, and no additional research-specific image enhancement or post-processing was performed during acquisition or archiving, in order to preserve real-world clinical imaging characteristics as much as possible. All CT examinations were routine clinical spinal scans reconstructed using conventional bone and soft-tissue windows, with slice thicknesses typically ranging from 1 to 3 mm.

There were certain differences in imaging equipment vendors and specific acquisition parameters across participating hospitals. These differences reflected the actual heterogeneity of multicenter clinical data. To reduce format-related differences in subsequent model processing, all images underwent unified field-of-view cropping, format conversion, and size standardization before entering the annotation and model training workflow.

### 2.3 Image annotation

X-ray image annotation in this study adopted a CT-referenced suspicious-region annotation strategy. These annotations do not represent precise lesion boundaries that can be independently determined on X-ray images alone. Instead, they provide suspicious-region localization references on X-ray images based on the CT-confirmed involved vertebral levels and their anatomical correspondence on radiographs. This strategy was used because the study focused on STB cases with equivocal X-ray findings, in which lesion boundaries are often difficult to determine using X-ray images alone, whereas CT can more clearly demonstrate osseous destruction, endplate erosion, vertebral margin irregularity, and adjacent soft-tissue changes.

During the annotation process, the involved vertebrae and adjacent levels were first identified on CT images. Suspicious regions were then localized on the corresponding X-ray images by referring to vertebral morphology, intervertebral spaces, endplate position, and the anatomical sequence of the spine. Based on the lesion levels, extent, and morphological characteristics observed on CT, the approximate projected regions on X-ray images were determined. Therefore, the annotation boxes in this study should be interpreted as CT-referenced suspicious regions on X-ray images, rather than precise ground-truth boundaries of X-ray-native lesions.

All X-ray annotations were completed using a web-based multimodal medical image annotation platform (version 2.6.4), which supports synchronized visualization of CT and X-ray images for cross-modality anatomical reference. Two radiologists with clinical experience independently annotated suspicious regions on X-ray images using rectangular bounding boxes. The annotation boxes typically covered one to two involved vertebral levels, depending on the CT-confirmed lesion extent and its projected appearance on X-ray images. For multilevel disease, the annotation box covered the overall suspicious projected region and adjacent structural changes to preserve the clinical integrity of the lesion region. When substantial discrepancies occurred in annotation location or extent, the two radiologists jointly reviewed the corresponding CT images and X-ray anatomical structures and reached a consensus annotation through discussion.

To further evaluate the potential cross-modality projection bias introduced by CT-referenced annotations, an agreement analysis between X-ray-only blinded annotations and CT-referenced annotations was additionally performed. Forty cases were randomly selected from the test set. For these cases, the annotator marked the most suspicious STB-related regions based only on X-ray images, without access to CT/MRI findings, original CT-referenced annotation boxes, or model predictions. The annotator was instructed to mark the most suspicious local abnormal regions on X-ray images; multiple bounding boxes were allowed if multiple suspicious regions were present, and no box was required if no localizable suspicious region could be identified.

The X-ray-only blinded annotation boxes were then compared with the original CT-referenced annotation boxes for spatial agreement. For each case, the maximum intersection over union (maxIoU) between all annotation boxes generated by the two annotation strategies was calculated as the spatial overlap metric. The proportions of cases with maxIoU ≥0.5 and maxIoU ≥0.3 were also calculated to reflect strict and loose spatial agreement, respectively. Wilson 95% confidence intervals were calculated for the X-ray-only annotatable rate and the agreement proportions at different maxIoU thresholds.

### 2.4 Dataset construction

The dataset used in this model was constructed from standard spinal X-ray images of the enrolled patients. All included cases were confirmed by CT or MRI to have STB-related structural lesions, and suspicious-region annotations were completed on the corresponding X-ray images using the CT-referenced suspicious-region annotation strategy. For cases in which the lesion extent involved multiple vertebral levels, an expanded annotation box was drawn to cover the relevant suspicious region according to its overall projected appearance on the X-ray image.

To prevent data leakage and ensure patient-level independence in model evaluation, dataset splitting was performed at the patient ID level. A total of 214 patients, approximately 70%, were randomly assigned to the training set, while the remaining 93 patients, approximately 30%, were allocated to the test set. Strict separation was enforced to ensure that images from the same patient did not appear in different data subsets. For patients with both anteroposterior and lateral X-ray images, all available projection views were included in model development and were assigned to the same data subset according to the patient ID–based splitting rule. This strategy avoided data leakage caused by different projection views from the same patient being distributed across the training and test sets.

Before model training and inference, images were resized using the default letterbox preprocessing strategy implemented in the Ultralytics framework. This strategy preserves the original aspect ratio while applying padding to match the required network input size. Training, testing, and inference were all performed using an input resolution of 640 × 640 pixels. It should be noted that letterbox resizing was used only for network input standardization and did not change the anatomical correspondence between the original annotation boxes and the suspicious regions.

During model training, multidimensional image preprocessing and data augmentation strategies were adopted for the single-class STB object detection task. All augmentation operations were performed online during the training phase only, while test images were not subjected to training augmentation in order to maintain consistency in performance evaluation. At the geometric level, mirror flipping, mild elastic deformation, random rotation, scale transformation, and resampling were applied to simulate variations in patient positioning, projection angle, and anatomical scale that may occur in clinical imaging. At the pixel level, brightness scaling, contrast adjustment, gamma transformation, and random noise were applied to simulate grayscale variations caused by different exposure conditions and imaging devices.

In addition to the standard augmentation workflow, a 256 × 256 center-crop operation was used during training as a local augmentation strategy to enhance the model's ability to learn subtle local structural changes. These cropped images were used only for data augmentation during training and were not used as the input format for model testing, inference, or deployment. During testing and the intended application stage, the model processed complete X-ray images after 640 × 640 letterbox resizing.

The data augmentation strategy followed common practice in medical object detection and was intended to improve model robustness to variations in patient positioning, anatomical scale, X-ray exposure parameters, and imaging-device heterogeneity. These augmentation operations were implemented using the Ultralytics framework, and this study did not conduct ablation experiments or separate optimization for individual augmentation parameters. Therefore, the data augmentation strategy itself was not regarded as an independent methodological innovation, but rather as a routine training procedure to improve model stability and generalization.

### 2.5 Model training strategy and evaluation metrics

This study adopted the YOLOv11 framework (6) as the base detection architecture. YOLOv11 is a one-stage object detection framework characterized by end-to-end prediction, relatively fast inference, and comparatively simple engineering deployment. This framework was selected mainly because of its mature implementation, training stability, computational efficiency, and potential applicability for real-time suspicious-region localization in resource-limited settings. It should be noted that this study did not perform a head-to-head benchmark between YOLOv11 and common detectors such as Faster R-CNN, RetinaNet, SSD, or EfficientDet; therefore, we do not claim that YOLOv11 is superior to other detection architectures for this task. The publicly available official Ultralytics implementation was used in this study without modification of the YOLOv11 network architecture.

Considering GPU memory limitations, the batch size was set to 4 to balance memory usage and training stability. For optimization, a learning rate scheduling strategy combining warm-up and linear annealing was used. The initial learning rate was set to 0.000651163. During the warm-up phase, the learning rate was gradually increased to this initial value to reduce parameter oscillations in early training. After warm-up, linear annealing was initiated from the fifth training epoch, and the learning rate decreased linearly as training progressed. The maximum number of training epochs was set to 50. An early stopping strategy was applied to improve training efficiency: training was terminated if the validation loss did not improve for 10 consecutive epochs.

During training, a validation subset was further split from the training set at the patient ID level for training monitoring, early stopping, and model selection. The test set was not involved in parameter updating, early stopping, or model selection, and was used only for final performance evaluation. This setting was intended to reduce the risk of data leakage and to ensure that test-set results reflected model performance on unseen patients.

The loss function followed the default multi-task loss formulation of the YOLOv11 detection model implemented in the Ultralytics framework, mainly including bounding box regression loss, classification loss, and distribution focal loss (DFL). The bounding box regression loss optimizes spatial matching between predicted boxes and reference annotation boxes, the classification loss is used for suspicious-region identification in the single-class object detection setting, and DFL improves the fine-grained accuracy of bounding box coordinate prediction. These loss components are jointly optimized through weighted summation to improve suspicious-region localization and object confidence prediction. Because this study used a single-class object detection setting, the classification output only indicates whether a candidate region corresponds to an STB-related suspicious region, and does not involve differential diagnosis between STB and non-tuberculous spinal diseases.

To evaluate model performance in the task of localizing STB-related suspicious regions with equivocal X-ray findings, standard metrics for single-class object detection were used, including mAP@0.5, mAP@0.5:0.95, precision, and recall. The mAP@0.5 metric evaluates the overall localization performance for reference suspicious regions at an IoU threshold of 0.5, whereas mAP@0.5:0.95 reflects localization stability under stricter spatial overlap requirements. In addition, precision-recall (PR) curves, precision-confidence curves, recall-confidence curves, and F1-confidence curves were analyzed to characterize model behavior across different confidence thresholds and the trade-off between precision and recall.

Considering that the intended use of the model is to provide early suspicious-region prompts for suspected cases with equivocal X-ray findings, rather than to exhaustively enumerate all lesions, patient-level detection rate and missed-case rate were further introduced as supplementary evaluation metrics. At a given confidence threshold, if at least one predicted box in a patient matched any reference suspicious-region annotation box with IoU ≥0.5, the case was regarded as successfully alerted. The patient-level detection rate was defined as the proportion of successfully alerted cases among all test cases, and the missed-case rate was defined as 1− patient-level detection rate. This metric reflects case-level alerting performance within the CT-confirmed STB-positive test cohort, rather than estimating specificity, referral yield, or false referral risk in real-world mixed clinical populations.

Based on the threshold-related performance analysis, a confidence threshold of 0.25 was used as the provisional working threshold in the current dataset to describe case-level performance in the context of early risk prompting and referral support. This threshold was selected because it lies in the low-to-moderate confidence range, allowing the model to retain more suspicious cases that may warrant further evaluation while avoiding excessive reliance on extremely low-confidence predictions. It should be emphasized that this threshold represents an exploratory operating point within the current positive test cohort and is not a final clinical deployment threshold. A clinically deployable threshold should be further determined in mixed clinical populations including normal cases and non-tuberculous spinal diseases through prospective validation or reader simulation studies.

## 3 Results

### 3.1 Training results

The performance of the YOLOv11 model in the task of localizing STB-related suspicious regions with equivocal X-ray findings is summarized in Table 1. Overall, the model showed relatively consistent detection performance across the training, validation, and test sets. The mAP@0.5 values on the training, validation, and test sets were 0.8129, 0.7686, and 0.7664, respectively, with only small differences among the data subsets. This result suggests that, under the current patient-level dataset split, the model maintained relatively stable CT-referenced suspicious-region localization performance, without obvious performance dissociation between the training and test sets. Considering that this study focused on STB-related regions with equivocal X-ray findings, poorly demarcated boundaries, and atypical structural changes, these results support the preliminary feasibility of localizing suspicious regions on X-ray images in CT-confirmed STB-positive cases.

**Table 1:** Performance evaluation of the YOLOv11 model for localizing STB-related suspicious regions with equivocal X-ray findings.

Dataset	mAP@0.5	mAP@0.5: 0.95	Precision	Recall
Training set	0.8129	0.5122	0.8440	0.6899
Validation set	0.7686	0.4783	0.8899	0.6129
Test set	0.7664	0.4520	0.8358	0.6215

Compared with mAP@0.5, the mAP@0.5:0.95 values under stricter IoU thresholds were relatively lower, with values of 0.5122, 0.4783, and 0.4520 for the training, validation, and test sets, respectively. This phenomenon is related to the imaging characteristics of the task and the annotation strategy used in this study. Early or mild STB manifestations on X-ray images often appear as low-contrast, poorly demarcated, or subtly altered structural regions, and this study used CT-referenced suspicious-region annotations established according to CT-confirmed lesion levels. Therefore, the decrease in localization performance under stricter IoU thresholds may partly reflect the spatial uncertainty caused by equivocal X-ray lesion boundaries and cross-modality projection-based annotation. This also indicates that the present results should be interpreted as localization performance for suspicious regions on X-ray images within CT-confirmed STB-positive cases, rather than as precise delineation of X-ray-native lesion boundaries.

The precision and recall values in Table 1 were used to summarize the overall detection performance of the model across the different data subsets. Because recall in object detection models varies with the confidence threshold, recall changes under different confidence thresholds are further reported in the following curve-based analysis to more fully describe model detection behavior.

Precision was higher than recall in all three data subsets. Across the training, validation, and test sets, precision remained within the range of 0.83–0.89, whereas recall was approximately 0.61–0.69. This result indicates that, in the current single-class suspicious-region localization task, the model output was relatively conservative and tended to retain candidate regions with higher confidence. It should be noted that, because this study did not include normal controls or non-tuberculous spinal disease controls, the precision reported in the table should not be interpreted as specificity, false-positive burden, or false referral risk in real-world mixed clinical populations.

The mAP@0.5 values of the validation and test sets were 0.7686 and 0.7664, respectively, showing close agreement. This result indicates that, under the current experimental setting in which multicenter data were pooled and split at the patient level, the model maintained performance on unseen test cases similar to that observed on the validation set. However, because data from all institutions were pooled before training, validation, and testing, explicit cross-center external generalization was not evaluated in this study.

### 3.2 Loss functions

**Figure 2:**
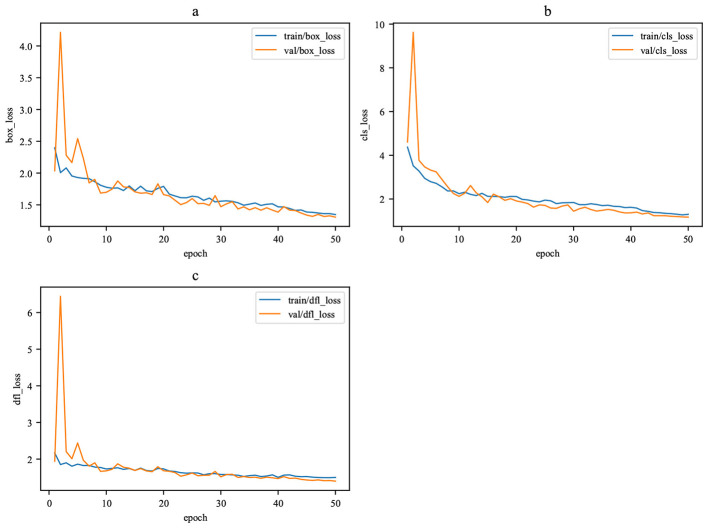
Loss function curves during model training. **(a)** Bounding box regression loss (box loss). **(b)** Classification loss (cls loss). **(c)** Distribution focal loss (DFL loss).

The changes in bounding box loss (box loss), classification loss (cls loss), and distribution focal loss (DFL loss) during model training are shown in Figure 2. Overall, all three loss components decreased during training, without persistent divergence or obvious oscillation. The training and validation loss curves showed generally consistent trends, suggesting basic stability of the training process.

It should be noted that the numerical relationship between training loss and validation loss should not be used alone to judge whether model training is effective. In object detection training, the training phase usually involves online data augmentation, regularization, and more complex sample perturbations, whereas validation is performed on relatively fixed data without strong augmentation. Therefore, validation loss being lower than or close to training loss does not necessarily indicate an error in the training process, but should instead be interpreted together with the loss trends, validation performance, and test performance. In this study, none of the loss components showed a late-stage rebound or persistent upward trend, and the mAP@0.5 values of the validation and test sets were close, suggesting that no obvious overfitting or training instability was observed.

Regarding the behavior of individual loss components, both the box loss and the DFL loss showed gradual decreases, indicating that the model progressively improved its spatial localization ability for CT-referenced suspicious regions during training. The classification loss also decreased and remained at a low level, suggesting that the model adapted to the current single-class object detection task. sHowever, because this study did not include non-tuberculous spinal disease controls or normal controls, the classification loss only reflects the model's ability to distinguish STB-related suspicious regions from background structures within the single-class detection framework, and cannot be used to infer differential diagnostic ability between tuberculous and non-tuberculous spinal lesions.

### 3.3 Curve-based analysis of model detection behavior

**Figure 3:**
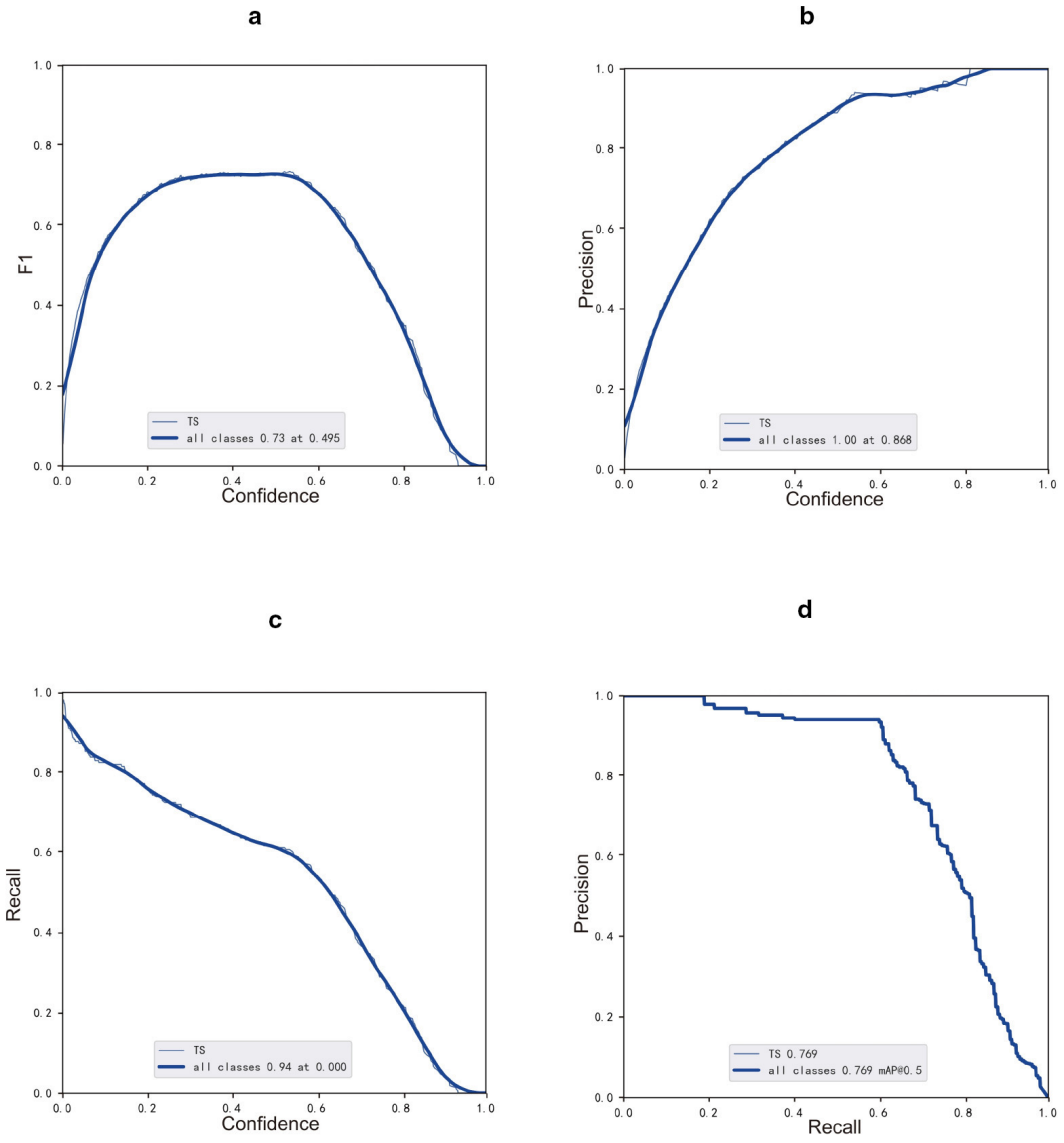
Comprehensive evaluation curves of the model's detection behavior on the test set. **(a)** F1-confidence curve. **(b)** Precision-confidence curve. **(c)** Recall-confidence curve. **(d)** Precision-recall curve.

F1-confidence, precision-confidence, recall-confidence, and precision-recall (PR) curves generated on the test set were used to further characterize the threshold-related prediction behavior of the model in the single-class CT-referenced suspicious-region localization task. The results are shown in Figure 3.

As shown in Figure 3a, the F1-confidence curve first increased, then entered a relatively stable range, and subsequently declined. Within a confidence range of approximately 0.35–0.55, the F1 score remained relatively stable, indicating that the model achieved a more balanced trade-off between precision and recall at moderate confidence thresholds. When the confidence threshold increased further, some low-confidence predictions that might still match the reference suspicious regions were filtered out, leading to decreased recall and a subsequent reduction in the F1 score.

As shown in Figure 3b, the precision-confidence curve generally increased as the confidence threshold increased. This trend is consistent with the common behavior of object detection models: higher confidence thresholds filter out more uncertain predictions, and the retained prediction boxes usually have higher matching reliability. It should be noted that the precision in this curve was still calculated within the current CT-confirmed STB-positive test set and reflects the matching between predicted boxes and CT-referenced suspicious regions. It should not be directly interpreted as specificity, false-positive burden, or false referral risk in real-world mixed clinical populations.

The recall-confidence curve shown in Figure 3c exhibited a decreasing trend. As the confidence threshold increased, the model discarded more low-confidence predictions, resulting in a gradual reduction in the number of reference suspicious regions that could be matched. This result indicates that threshold selection directly affects suspicious-region-level detection ability. A higher threshold can improve the matching reliability of prediction boxes, but at the cost of reduced recall.

As shown in Figure 3d, the PR curve further illustrates the trade-off between precision and recall under different operating points. The model maintained relatively high precision in the low-to-moderate recall range, whereas precision decreased markedly when recall was further increased. This curve pattern suggests that, to capture more suspicious regions with equivocal X-ray findings, the model needs to retain more low-confidence predictions, which introduces more uncertain localization results. Therefore, the selection of the confidence threshold should be interpreted in relation to the intended use of the model as an early risk prompting and referral support tool, rather than relying on a single scalar metric alone.

In addition, the above curves mainly reflect threshold-related detection behavior at the reference suspicious-region level. For referral prompting scenarios, a more directly relevant question is whether the model can provide at least one effective suspicious-region prompt for a given case. Therefore, patient-level detection analysis was further performed in the following section to evaluate the model's case-level alerting performance.

### 3.4 Patient-level detection analysis for referral prompting

**Figure 4:**
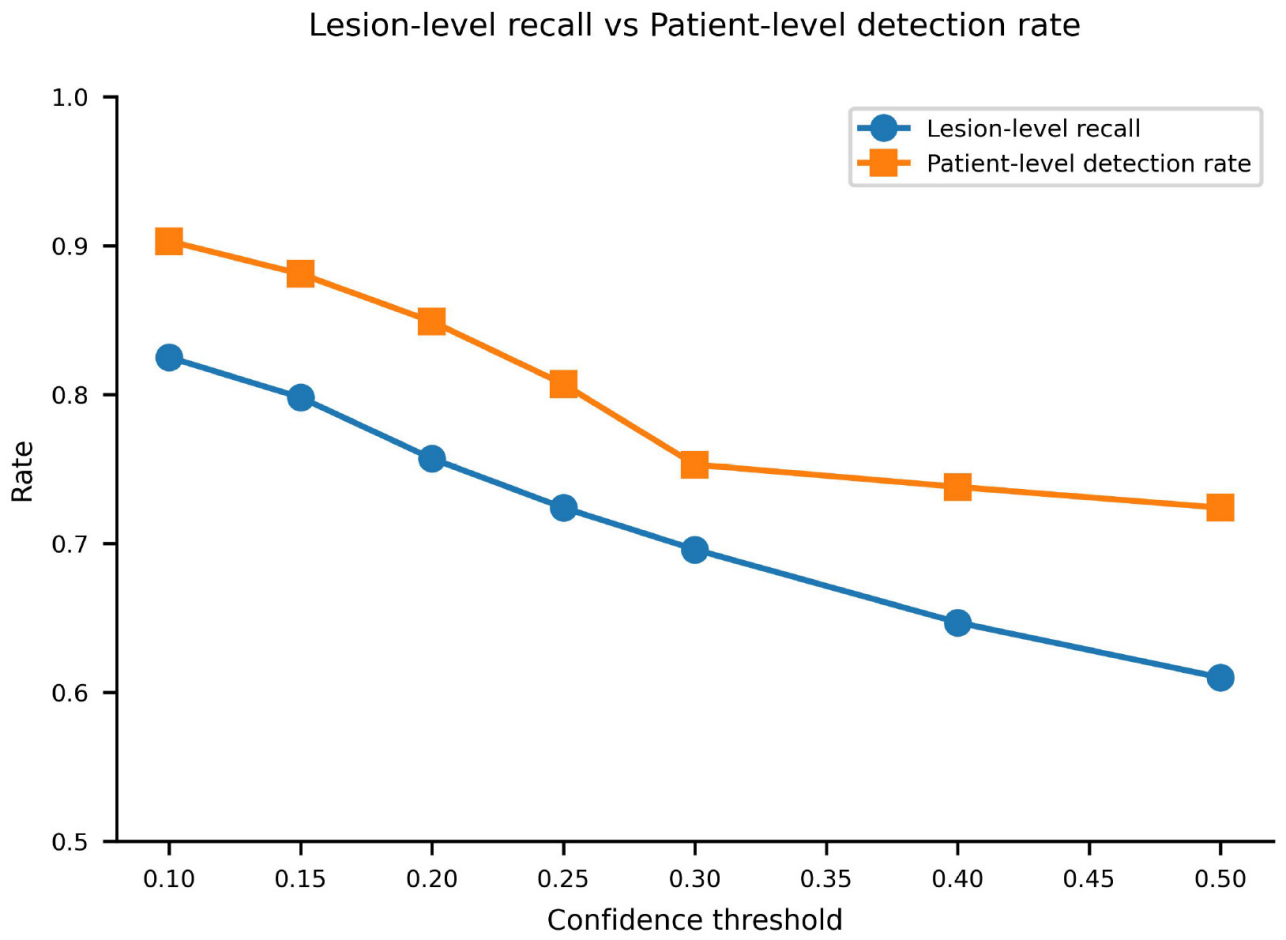
Comparison between region-level recall and patient-level detection rate under different confidence thresholds. Patient-level detection rate was defined as the proportion of cases in the fixed test set in which at least one predicted box matched any reference suspicious-region annotation box with IoU ≥ 0.5.

Considering that the intended use of the model is not to exhaustively enumerate all suspicious regions, but to provide early suspicious-region prompts in cases with equivocal X-ray findings to support further imaging evaluation or referral decisions, patient-level detection analysis was further performed. Unlike region-level recall, which focuses on the proportion of all reference suspicious regions that are successfully detected, patient-level detection rate focuses on whether the model can provide at least one effective prompt for a given case. It was defined as follows: at a given confidence threshold, if at least one predicted box in a patient matched any reference suspicious-region annotation box in that patient with IoU ≥0.5, the case was regarded as successfully alerted. The comparison between region-level recall and patient-level detection rate under different confidence thresholds is shown in Figure 4.

As the confidence threshold increased from 0.10 to 0.50, region-level recall decreased from 0.8251 to 0.6104, while patient-level detection rate decreased from 0.9032 to 0.7247. Both metrics showed a decreasing trend. This result indicates that higher confidence thresholds cause the model to discard more uncertain predictions, thereby reducing both region-level and patient-level detection ability.

Notably, patient-level detection rate was higher than region-level recall at all confidence thresholds. At the threshold of 0.25, the patient-level detection rate was 0.8073, whereas the region-level recall was 0.7244. This result indicates that, although the model may not completely detect all reference suspicious regions within the same case, it can still provide at least one suspicious-region prompt in most cases. For referral prompting scenarios, this case-level alerting ability is more closely aligned with the primary-care decision of whether further imaging evaluation is warranted than region-level recall alone.

Based on the above threshold analysis, a confidence threshold of 0.25 was used as the provisional working threshold in the current dataset to describe case-level performance in the referral prompting scenario. At this threshold, the patient-level detection rate was 0.8073, corresponding to a missed-case rate of 0.1927. Compared with higher thresholds, this threshold retained more suspicious-case prompts; compared with lower thresholds, it avoided excessive reliance on extremely low-confidence predictions. Therefore, 0.25 can be regarded as an exploratory operating point for case-level alerting analysis in this study. It should be emphasized that this patient-level analysis was still performed within the CT-confirmed STB-positive test cohort, and this threshold should not be regarded as a final clinical deployment threshold. It cannot be used to infer referral yield, specificity, false referral risk, or real-world referral rate in mixed clinical populations.

### 3.5 Agreement analysis between X-ray-only blinded annotations and CT-referenced annotations

To further evaluate the spatial correspondence between CT-referenced annotations and suspicious regions on X-ray images, an agreement analysis between X-ray-only blinded annotations and CT-referenced annotations was additionally performed. Forty cases were randomly selected from the test set. For these cases, the annotator marked the most suspicious STB-related regions based only on X-ray images, without reference to CT/MRI findings, original CT-referenced annotation boxes, or model predictions. The X-ray-only blinded annotations were then compared with the original CT-referenced annotations, and the spatial agreement between the two annotation strategies was calculated. The results are shown in Table 2.

**Table 2:** Spatial agreement analysis between X-ray-only blinded annotations and CT-referenced annotations.

Metric	Value	95% confidence interval
Number of analyzed cases	40	–
X-ray-only annotatable cases	40/40 (100.0%)	91.2%–100.0%
Mean maximum IoU	0.7542	–
Cases with maxIoU ≥0.5	34/40 (85.0%)	70.9%–92.9%
Cases with maxIoU ≥0.3	38/40 (95.0%)	83.5%–98.6%

Among the 40 randomly selected test cases, the X-ray-only annotatable rate was 100.0%, indicating that, for cases with equivocal X-ray findings retained after the preceding screening process, the annotator could still identify at least one suspicious region based on X-ray images alone. This result is consistent with the case selection logic of this study: the final included cases were not completely negative or invisible on X-ray images, but rather showed suspicious clues that were insufficient for a definitive diagnosis.

The mean maximum IoU between X-ray-only blinded annotations and CT-referenced annotations was 0.7542, indicating relatively good spatial agreement between the two annotation strategies. Furthermore, 34/40 cases reached a maxIoU ≥0.5, corresponding to an agreement rate of 85.0%, and 38/40 cases reached a maxIoU ≥0.3, corresponding to an agreement rate of 95.0%. These results suggest that, in most cases with equivocal X-ray findings, CT-referenced annotation regions showed good spatial correspondence with suspicious regions identified by X-ray-only blinded annotation. Therefore, CT-referenced annotations were not completely detached from suspicious imaging clues visible on X-ray images; rather, they used CT findings to provide more refined anatomical localization on the basis of already existing equivocal abnormalities on X-ray images.

In terms of annotation extent, X-ray-only blinded annotations generally tended to cover broader suspicious areas, whereas CT-referenced annotation boxes were more concentrated and more closely aligned with CT-confirmed involved lesion levels. Therefore, this supplementary analysis supports relatively good spatial agreement between CT-referenced annotations and suspicious regions on X-ray images, but it cannot completely exclude cross-modality projection bias introduced by CT-referenced annotation. The model output in this study should still be interpreted as CT-referenced suspicious-region localization on X-ray images, rather than precise delineation of X-ray-native lesion boundaries.

### 3.6 Visualization and case analysis

**Figure 5:**
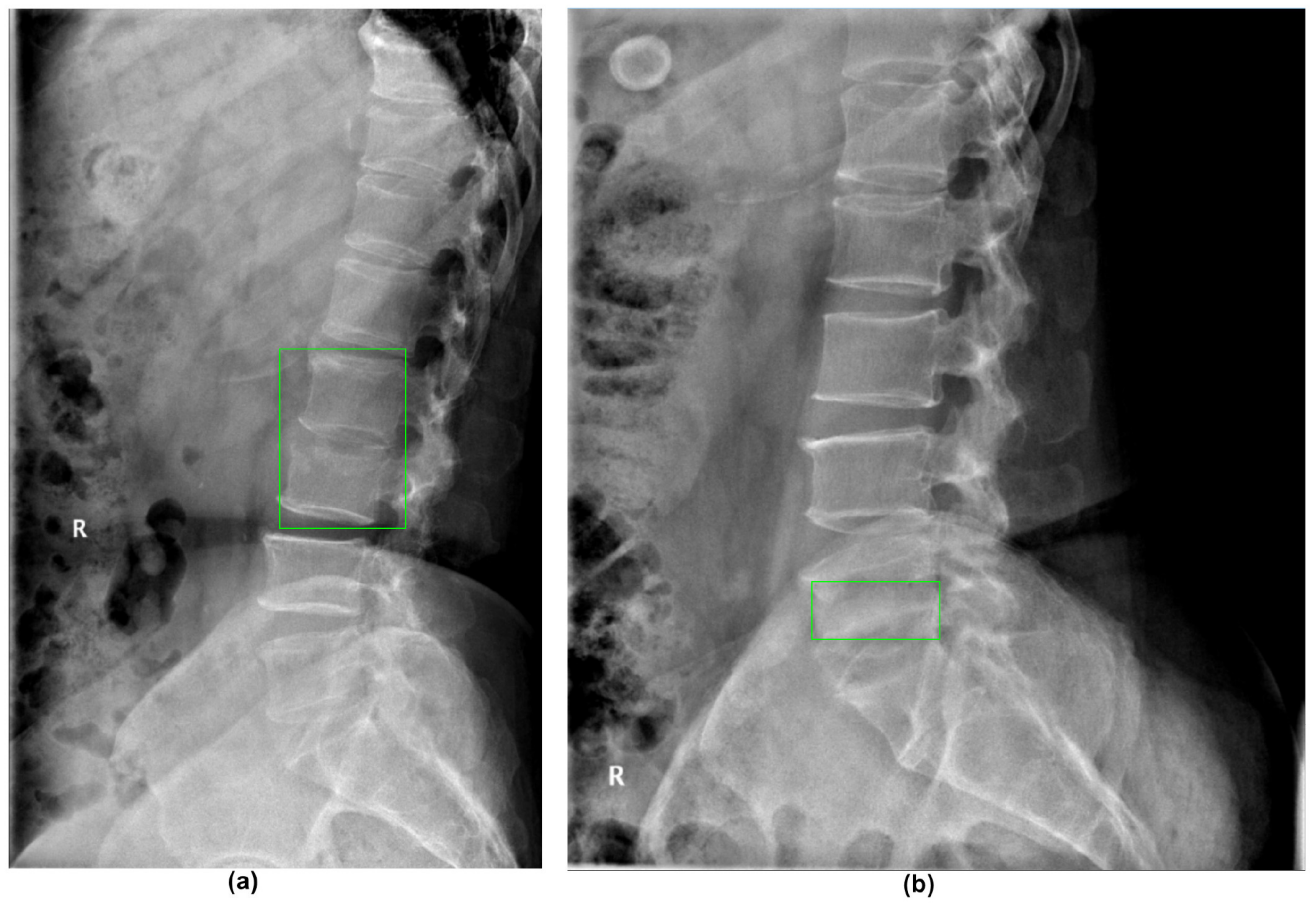
Examples of suspicious-region prompts generated by the YOLOv11 model in two spinal tuberculosis cases with equivocal X-ray findings. **(a)** Example showing a suspicious region spanning multiple adjacent vertebral levels. **(b)** Example showing a more localized suspicious region. Green boxes indicate the suspicious regions predicted by the YOLOv11 model.

Figure 5 presents two representative cases in which the model provided suspicious-region prompts. The prompted regions were located in the mid-to-lower lumbar spine and the lumbosacral junction, respectively, reflecting typical scenarios with different anatomical levels and imaging complexity. Both patients presented with lower back-related symptoms, and their lateral X-ray images did not show typical late-stage signs of spinal tuberculosis. Instead, the images showed only low-contrast, poorly demarcated, or mildly altered structural regions. Therefore, a definitive diagnosis based on X-ray images alone was difficult, and these cases belonged to the equivocal X-ray finding category targeted in this study.

In the case shown in Figure 5a, the patient was a 70-year-old male whose X-ray image showed only mild irregularity of the vertebral body contour and subtle intervertebral space changes, without typical signs of osseous destruction. In this case, the YOLOv11 model generated a suspicious-region prompt in the mid-to-lower lumbar spine (green bounding box). Subsequent CT examination confirmed vertebral body destruction, endplate erosion and sclerosis, and adjacent paravertebral soft-tissue involvement at the corresponding level. The CT-confirmed involved level showed good spatial correspondence with the region prompted by the model on the X-ray image.

The case shown in Figure 5b illustrates the difficulty of localizing lesions at the lumbosacral junction on X-ray images. The patient was a 43-year-old female whose X-ray image showed a largely preserved spinal structure, with only mild changes in the intervertebral space and endplate region. Such findings may be confused with nonspecific imaging appearances such as degenerative changes. In this case, the model generated a suspicious-region prompt at the L5–S1 level. CT examination demonstrated infectious changes in the L5 vertebral body accompanied by formation of a left-sided psoas abscess, consistent with STB-related imaging findings. The region prompted by the model showed good spatial correspondence with the CT-confirmed involved level.

These visualization results further indicate that the model can provide suspicious-region prompts corresponding to CT-confirmed levels in some cases with equivocal X-ray findings. However, these examples are intended only to demonstrate the spatial correspondence between model outputs and CT-referenced regions. They do not replace systematic diagnostic performance evaluation and should not be used to infer the model's differential diagnostic ability in normal cases or non-tuberculous spinal diseases.

## 4 Discussion

### 4.1 Main findings and study positioning

Existing intelligent imaging studies on spinal tuberculosis and related spinal infections have mainly focused on cross-sectional imaging modalities such as CT or MRI. These modalities can more clearly depict osseous destruction, endplate erosion, paravertebral soft-tissue involvement, and bone marrow edema, and are therefore more suitable for differential diagnosis between spinal tuberculosis and other infectious, neoplastic, or degenerative spinal diseases (7–13, 17, 21). In contrast, identifying STB-related abnormalities based only on X-ray images is more challenging, especially in early-stage or mild disease, where radiographic findings are often characterized by low contrast, poorly demarcated boundaries, or subtle structural changes. Existing X-ray-based deep learning studies have more commonly focused on chest X-ray tuberculosis screening or other bone disease classification tasks. Systematic object detection research remains limited for the referral prompting scenario in primary-care settings, where X-ray findings are equivocal but clinical suspicion of STB persists (14, 15).

Based on 307 cases meeting the criterion of equivocal X-ray findings but CT-confirmed STB positivity, this study constructed and validated a single-class object detection model for suspicious-region localization on X-ray images. On the test set, the model achieved an mAP@0.5 of 0.7664, and the mAP@0.5 values of the validation and test sets were close, suggesting relatively stable CT-referenced suspicious-region localization performance under the current patient-level dataset split. It should be emphasized that this result should be interpreted as suspicious-region localization performance within CT-confirmed STB-positive cases, rather than diagnostic performance in mixed clinical populations. Because this study did not include normal controls or non-tuberculous spinal disease controls, the current results cannot be used to infer model specificity, false-positive burden, or differential diagnostic ability in real-world mixed clinical populations.

From the perspective of intended use, this study did not aim to establish a screening system for all patients with back pain or the general population, nor to build an automated differential diagnostic model for distinguishing STB from non-tuberculous spinal diseases. Instead, this study focused on a narrower problem in primary-care or resource-limited medical settings: when clinicians have developed clinical suspicion of STB based on symptoms, medical history, or initial imaging findings, but the X-ray findings remain equivocal and insufficient for a definitive diagnosis, can the model prompt suspicious regions on X-ray images corresponding to CT-confirmed involved levels? Therefore, the intended target cases are neither radiographically normal cases nor typical late-stage cases that can be clearly diagnosed on plain radiographs alone, but the intermediate group of suspicious yet indeterminate cases that most often create uncertainty in primary-care interpretation. In this sense, the present study should be positioned as a proof-of-concept feasibility investigation. Its main contribution is to demonstrate that a deep learning model may provide CT-referenced suspicious-region prompts at the equivocal X-ray stage, thereby offering supportive information for further CT/MRI examination or referral decisions.

Therefore, the appropriate interpretive boundary of the proposed model should be limited to early risk prompting and referral support, rather than independent diagnosis. The bounding boxes generated by the model are intended to highlight suspicious regions on X-ray images for clinicians to interpret together with symptoms, laboratory findings, and subsequent advanced imaging results. This positioning is consistent with the primary-care workflow of first identifying suspicious clues and then referring patients for confirmatory evaluation. However, its real-world clinical utility still requires further validation in prospective studies involving mixed cases, including normal subjects, degenerative spinal diseases, spinal tumors, and non-tuberculous spinal infections (18).

### 4.2 Patient-level alerting ability and threshold interpretation

Conventional object detection metrics mainly reflect model performance at the level of reference suspicious-region localization, but they are not fully equivalent to the clinical decision problem in referral prompting scenarios. For early risk prompting in primary care, the key question is not whether the model can exhaustively enumerate all suspicious regions within the same case, but whether it can provide at least one effective prompt for a given case, thereby facilitating further CT/MRI examination or referral assessment. Therefore, relying only on region-level metrics such as mAP, precision, and recall may be insufficient to reflect the case-level role of the model as a referral support tool.

Based on this consideration, patient-level detection rate and missed-case rate were further introduced as supplementary evaluation metrics. Patient-level detection rate was defined as follows: at a given confidence threshold, if at least one predicted box in a case matched any reference suspicious-region annotation box with IoU ≥0.5, the case was considered successfully alerted. The missed-case rate was defined as 1− patient-level detection rate. This metric is more closely aligned with the practical question in referral prompting scenarios, namely whether the model can provide at least one imaging region worthy of further attention for a suspected case.

The results showed that patient-level detection rate was higher than region-level recall at all evaluated thresholds. When the confidence threshold increased from 0.10 to 0.50, region-level recall decreased from 0.8251 to 0.6104, whereas patient-level detection rate decreased from 0.9032 to 0.7247, corresponding to an increase in missed-case rate from 0.0968 to 0.2753. This trend indicates that, as the threshold increases, the model discards more low-confidence predictions, thereby reducing both region-level and case-level detection ability. At the same time, patient-level detection rate remained consistently higher than region-level recall, suggesting that even when the model does not completely detect all reference suspicious regions within the same case, it may still provide at least one effective suspicious-region prompt for that case.

Regarding threshold selection, a confidence threshold of 0.25 was used as the provisional working threshold in the current dataset to describe case-level model performance in the referral prompting scenario. At this threshold, the patient-level detection rate was 0.8073, corresponding to a missed-case rate of 0.1927, while the region-level recall was 0.7244. This threshold was selected because it lies within a low-to-moderate confidence range, allowing more suspicious-case prompts to be retained while avoiding complete reliance on extremely low-confidence predictions. Therefore, 0.25 should be regarded as an exploratory operating point in the positive test cohort of this study, rather than a final threshold optimized for clinical deployment.

It should be emphasized that the current patient-level detection rate was still calculated within the CT-confirmed STB-positive test cohort. Because this study did not include normal controls or non-tuberculous spinal disease controls, it cannot estimate the real-world referral rate, false referral burden, specificity, or referral yield in mixed clinical populations. Therefore, patient-level detection rate can only be used to describe the model's case-level alerting ability within positive cases, and should not be equated with referral yield or decision-curve net benefit in real-world primary-care settings. Future studies should determine a more appropriate operating threshold for clinical deployment in mixed cohorts including normal cases and disease-mimicking conditions, combined with decision-curve analysis, reader simulation, or prospective referral studies.

### 4.3 CT-referenced annotation and cross-modality annotation bias

This study used CT-referenced suspicious-region annotations as the spatial reference for model training and evaluation. This strategy was necessary because the enrolled cases had equivocal X-ray findings but were CT-confirmed STB-positive, and lesion boundaries or involved levels are often difficult to determine reliably based on X-ray images alone. CT can more clearly demonstrate osseous destruction, endplate erosion, vertebral margin irregularity, and adjacent soft-tissue involvement, thereby providing anatomical reference for suspicious-region localization on X-ray images. Compared with purely subjective annotation based only on X-ray images, CT-referenced annotation can reduce annotation uncertainty caused by equivocal lesion boundaries and improve spatial consistency between annotators (16).

However, CT-referenced annotation may also introduce methodological bias. Because projection differences exist between two-dimensional X-ray images and three-dimensional CT structures, the corresponding regions of CT-confirmed lesion levels on X-ray images do not necessarily have clear and independently visible boundaries. Therefore, CT-referenced annotation boxes may partly reflect spatial priors provided by CT, rather than fully corresponding to X-ray-native lesion boundaries visible on radiographs. This cross-modality projection uncertainty may affect the model training target and the interpretation of performance, especially under stricter IoU thresholds, where small spatial offsets between predicted boxes and reference boxes can lead to decreases in evaluation metrics. Therefore, the model output in this study should be interpreted as CT-referenced suspicious-region localization on X-ray images, rather than precise delineation of X-ray-native lesion boundaries.

To further evaluate the spatial correspondence between CT-referenced annotations and suspicious regions on X-ray images, this study additionally performed an agreement analysis between X-ray-only blinded annotations and CT-referenced annotations. The results showed that, among 40 randomly selected test cases, the annotator could identify at least one suspicious region based on X-ray images alone in all cases, corresponding to an X-ray-only annotatable rate of 100.0%. The mean maximum IoU between X-ray-only blinded annotations and CT-referenced annotations was 0.7542. Among these cases, 34/40 reached a maxIoU ≥0.5, corresponding to an agreement rate of 85.0%, and 38/40 reached a maxIoU ≥0.3, corresponding to an agreement rate of 95.0%. These results suggest that, among cases with equivocal X-ray findings retained after the preceding multi-reader screening process, CT-referenced annotation regions showed good spatial correspondence with suspicious regions identified by X-ray-only blinded annotation. In other words, CT-referenced annotations were not completely detached from suspicious imaging clues visible on X-ray images; rather, they used CT findings to provide more refined anatomical localization on the basis of already existing equivocal abnormalities on X-ray images.

At the same time, this supplementary analysis cannot prove that CT-referenced annotation is completely unbiased. In the actual annotation process, X-ray-only blinded annotations generally tended to cover broader suspicious areas, whereas CT-referenced annotation boxes were more concentrated and more closely aligned with CT-confirmed lesion levels. This difference indicates that, although the two annotation strategies showed good spatial agreement, measurable differences in annotation extent and cross-modality projection uncertainty still remained. Therefore, the current results should not be interpreted as evidence that the model has learned lesion boundaries that are completely and independently visible on X-ray images. Rather, they should be understood as showing that the model can prompt suspicious regions on X-ray images that correspond to CT-referenced regions in CT-confirmed STB-positive cases.

Future studies should further expand the scale of agreement analysis between X-ray-only blinded annotations and CT-referenced annotations, and should include multiple radiologists with different levels of experience to quantify annotation differences across readers, lesion sizes, and spinal levels. In particular, further analysis is needed to determine whether annotation agreement is affected by lesion size, involved vertebral level, projection view, and lesion location. Such analyses will help more accurately evaluate the reliability of CT-referenced annotations and clarify the applicability boundaries of model performance under different imaging visibility conditions.

### 4.4 Model selection and interpretability

This study adopted YOLOv11 as the base object detection framework, mainly because of its one-stage detection structure, inference efficiency, mature open-source implementation, and potential applicability for real-time suspicious-region prompting in resource-limited settings. Compared with two-stage detectors such as Faster R-CNN, the YOLO series generally has a more streamlined inference process and higher computational efficiency. Compared with common detection frameworks such as RetinaNet, SSD, and EfficientDet, YOLOv11 provides good operability in engineering deployment and training implementation. However, this study did not perform a head-to-head benchmark among different detectors; therefore, the results should not be interpreted as evidence that YOLOv11 is superior to other object detection architectures for this task. Future studies should compare different detectors under the same dataset split and evaluation criteria to further clarify the influence of model architecture selection on suspicious-region localization performance for STB cases with equivocal X-ray findings (19).

In terms of output format, object detection boxes can directly indicate suspicious regions on X-ray images, and therefore provide more intuitive spatial interpretability than case-level classification results alone. Such visual outputs may help clinicians focus on vertebral levels that may require further evaluation. However, detection boxes only reflect the final spatial outputs of the model and cannot fully reveal which textures, edges, intervertebral space changes, or cortical bone abnormalities the model relies on during inference. Future research may incorporate saliency analysis methods suitable for object detection models, such as box-specific Grad-CAM, Eigen-CAM, or other detection-specific saliency analyses, to further evaluate the consistency between model-attended regions and suspicious signs identified by radiologists (20).

### 4.5 Limitations and future work

This study has several limitations. First, the study cohort included only CT-confirmed STB-positive cases with equivocal X-ray findings, without normal controls or non-tuberculous spinal disease controls. Therefore, the current results mainly reflect CT-referenced suspicious-region localization ability within CT-confirmed STB-positive cases and cannot be used to estimate specificity, false-positive burden, false referral risk, or real-world referral yield in mixed clinical populations. Future studies should include normal cases, degenerative spinal diseases, spinal tumors, non-tuberculous spinal infections, and other conditions that may mimic STB imaging findings to further evaluate model performance in real-world primary-care referral scenarios.

Second, the patient-level detection rate and missed-case rate proposed in this study can supplement region-level detection metrics, but this analysis was still performed within the CT-confirmed STB-positive test cohort. Therefore, patient-level detection rate should not be equated with referral yield in real-world mixed clinical populations. This study used a confidence threshold of 0.25 as the provisional working threshold in the current positive test cohort to describe case-level alerting ability, but this threshold is not a final clinical deployment threshold. A real deployment threshold should be further determined in mixed cohorts including normal cases and disease-mimicking conditions, combined with reader studies, decision-curve analysis, or prospective referral studies.

Third, although this study presented an agreement analysis between X-ray-only blinded annotations and CT-referenced annotations, CT-referenced annotation may still involve cross-modality projection bias. There are unavoidable projection differences between two-dimensional X-ray images and three-dimensional CT structures, and the corresponding regions of CT-confirmed lesions on X-ray images may not always have clear and independently visible boundaries. Therefore, the model output in this study should be interpreted as CT-referenced suspicious-region localization on X-ray images, rather than precise delineation of X-ray-native lesion boundaries. Future studies may further expand the sample size for X-ray-only blinded annotation and combine multi-reader annotation analysis to evaluate annotation consistency across different lesion sizes, spinal levels, and projection views.

Fourth, although the data in this study were collected from multiple hospitals, data from all centers were pooled before training, validation, and testing, and explicit site-wise external validation was not performed. Therefore, the generalizability of the model to new medical institutions, different imaging devices, or different patient spectra remains to be further validated. In addition, this was a retrospective study, and paired X-ray and CT/MRI examinations may have involved certain time intervals. Future prospective studies should further control the imaging acquisition workflow to improve the consistency of cross-modality reference standards.

Finally, this study has not yet been prospectively validated in real primary-care environments. The current results support the technical feasibility of providing suspicious-region prompts on X-ray images for CT-confirmed STB-positive cases, but the actual impact of the model on primary-care referral decisions, examination delays, and healthcare resource utilization still needs to be evaluated in real clinical workflows.

## 5 Conclusion

This study addressed the need for early prompting of spinal tuberculosis cases with equivocal X-ray findings in primary-care settings. Based on 307 CT-confirmed STB cases, a YOLOv11-based single-class object detection model was developed for suspicious-region localization on X-ray images. Using patient-level data splitting and model evaluation, the model achieved an mAP@0.5 of 0.7664 on the test set, with similar performance between the validation and test sets. These results indicate that, under the current data conditions, the model can provide relatively stable localization prompts for STB-related suspicious regions on X-ray images.

To account for the difference between conventional object detection metrics and referral prompting scenarios, this study further evaluated model performance from a case-level perspective. At the provisional working threshold of 0.25, the patient-level detection rate was 0.8073 and the missed-case rate was 0.1927, indicating that the model could provide at least one effective suspicious-region prompt in most CT-confirmed STB-positive cases. Compared with region-level recall alone, patient-level detection rate is more closely aligned with the practical primary-care question of whether further imaging evaluation or referral is warranted.

In addition, this study performed an agreement analysis between X-ray-only blinded annotations and CT-referenced annotations. The results showed that all 40 randomly selected test cases could be annotated with at least one suspicious region based on X-ray images alone. The mean maximum IoU between X-ray-only blinded annotations and CT-referenced annotations was 0.7542, and 85.0% of cases reached a maxIoU ≥0.5. These findings indicate that, among the screened cases with equivocal X-ray findings, CT-referenced annotation regions showed good spatial correspondence with suspicious imaging clues on X-ray images, providing supplementary support for the rationale of the CT-referenced suspicious-region annotation strategy.

Overall, this study demonstrates that, using X-ray images alone as model input, a deep learning model can provide suspicious-region localization prompts for CT-confirmed STB-positive cases with equivocal X-ray findings. The proposed approach provides a feasible technical pathway for X-ray-based early risk prompting and referral support for spinal tuberculosis in resource-limited settings. Further studies should include normal cases and non-tuberculous spinal disease controls, and should conduct external validation and prospective clinical evaluation to further assess model applicability in real primary-care settings.

## Data Availability

The imaging datasets generated and analyzed during this study are not publicly available due to ethical restrictions and patient privacy concerns. They consist of clinical medical images containing potentially identifiable human information. Access to the de-identified data may be granted to qualified researchers for legitimate scientific purposes upon reasonable request to the corresponding author, subject to approval by the relevant Institutional Review Boards and the execution of a formal data use and confidentiality agreement. Requests to access these datasets should be directed to corresponding author: Mayidili Nijiati: mydl0911@163.com.
